# Supplementary material for: Synchrotron intensity gradient revealing magnetic fields in galaxy clusters
Source: Nat Commun. 2024 Feb 6;15:1006. doi: 10.1038/s41467-024-45164-8 (PMC10847451; doi:10.1038/s41467-024-45164-8)
Supplement: Supplementary file 1 — Supplementary Information [file 41467_2024_45164_MOESM1_ESM.pdf]

Synchrotron Intensity Gradient Revealing Magnetic Fields in Galaxy Clusters

Yue Hu[1,2,*], C. Stuardi[3,4], A. Lazarian[2,*], G. Brunetti[4], A. Bonafede[3,4], and Ka Wai Ho[2,5]

[1]Department of Physics, University of Wisconsin-Madison, Madison, WI 53706, USA
[2]Department of Astronomy, University of Wisconsin-Madison, Madison, WI 53706, USA
[3]Dipartimento di Fisica e Astronomia, Università di Bologna, via Gobetti 93/2, I-40129 Bologna, Italy
[4]INAF - Istituto di Radioastronomia di Bologna, Via Gobetti 101, I-40129 Bologna, Italy
[5]Theoretical Division, Los Alamos National Laboratory, Los Alamos, NM 87545, USA
[*]e-mail: yue.hu@wisc.edu, alazarian@facstaff.wisc.edu

Supplementary Information

Low-resolution magnetic field maps in RXC J1314.4 - 2515

Supplementary Fig. 1 displays the low-resolution magnetic field maps in RXC J1314.4 - 2515 determined through SIG and synchrotron polarization. It provides a comparison with the high-resolution SIG measurement in Main Text Fig. 1. The high-resolution SIG measurement in RXC J1314.4 - 2515 (FWHM around 120 kpc) eliminates the resolution difference and shows better agreement with synchrotron polarization compared to the low-resolution measurement (FWHM around 240 kpc).

Uncertainty of the magnetic field direction measured by the SIG

The magnetic field mapped by the SIG method is subject to two sources of uncertainty: (1) systematic errors in the observational map and (2) the uncertainty inherent in the SIG algorithm itself. The latter uncertainty arises from the subregion-fitting approach utilized in the algorithm. Specifically, the SIG fits a Gaussian histogram to the orientation of the gradient within a subregion and outputs the angle corresponding to the peak value of the histogram. The associated uncertainty can be quantified as the error $\sigma_{\psi_{\rm s}}(x,y)$ of the Gaussian fitting algorithm within a confidence level 95%.

Considering the noise $\sigma_{\rm I}(x,y)$, which is assumed to be constant, in intensity map $I(x,y)$ and error propagation, the uncertainties $\sigma_q(x,y)$ and $\sigma_u(x,y)$ of the Pseudo Stokes parameters $Q_{\rm g}(x,y)$ and $U_{\rm g}(x,y)$ can be obtained from:

$$\sigma_{\cos}(x,y) = |2\sin(2\psi_{\rm s}(x,y))\sigma_{\psi_{\rm s}}(x,y)|, \tag{1}$$

$$\sigma_{\sin}(x,y) = |2\cos(2\psi_{\rm s}(x,y))\sigma_{\psi_{\rm s}}(x,y)|, \tag{2}$$

$$\sigma_q(x,y) = |I\cdot\cos(2\psi_{\rm s})|\sqrt{(\frac{\sigma_{\rm I}}{I})^2+(\frac{\sigma_{\cos}}{\cos(2\psi_{\rm s})})^2}, \tag{3}$$

$$\sigma_u(x,y) = |I\cdot\sin(2\psi_{\rm s})|\sqrt{(\frac{\sigma_{\rm I}}{I})^2+(\frac{\sigma_{\sin}}{\sin(2\psi_{\rm s})})^2}, \tag{4}$$

$$\sigma_{\psi_{\rm g}}(x,y) = \frac{|\frac{U_{\rm g}}{Q_{\rm g}}|\sqrt{(\frac{\sigma_{\rm q}}{Q_{\rm g}})^2+(\frac{\sigma_{\rm u}}{U_{\rm g}})^2}}{2[1+(\frac{U_{\rm g}}{Q_{\rm g}})^2]}, \tag{5}$$

where $\sigma_{\psi_{\rm g}}(x,y)$ gives the angular uncertainty of the resulting magnetic field direction. Uncertainty maps are presented in Supplementary Figs. 2 and 3. The median value is listed in Main Text Tab. 2.

In addition to the signal uncertainty, other physical mechanisms may also have contributed. For instance, the shock front can provide extra gradients, which are not associated with turbulence, but may contribute to the total intensity gradient. In the case of perpendicular shocks, the rapid jump in intensity at the shock front creates an intensity gradient perpendicular to the magnetic field. This relationship between the orientation of the intensity gradient and magnetic field remains true except in the rare case of parallel shock, which is typically not observed in weakly magnetized media[1]. However, it is important to note that, in radio relics, SIG is actually measuring the magnetic field at the shock downstream regions, in which the jump of intensity does not appear, so SIG still works there. As for the shock front, it is typically a very thin strip that occupies only one observational beam. The sub-block averaging (over a number of beams) method adopted in SIG, on the other hand, diminishes

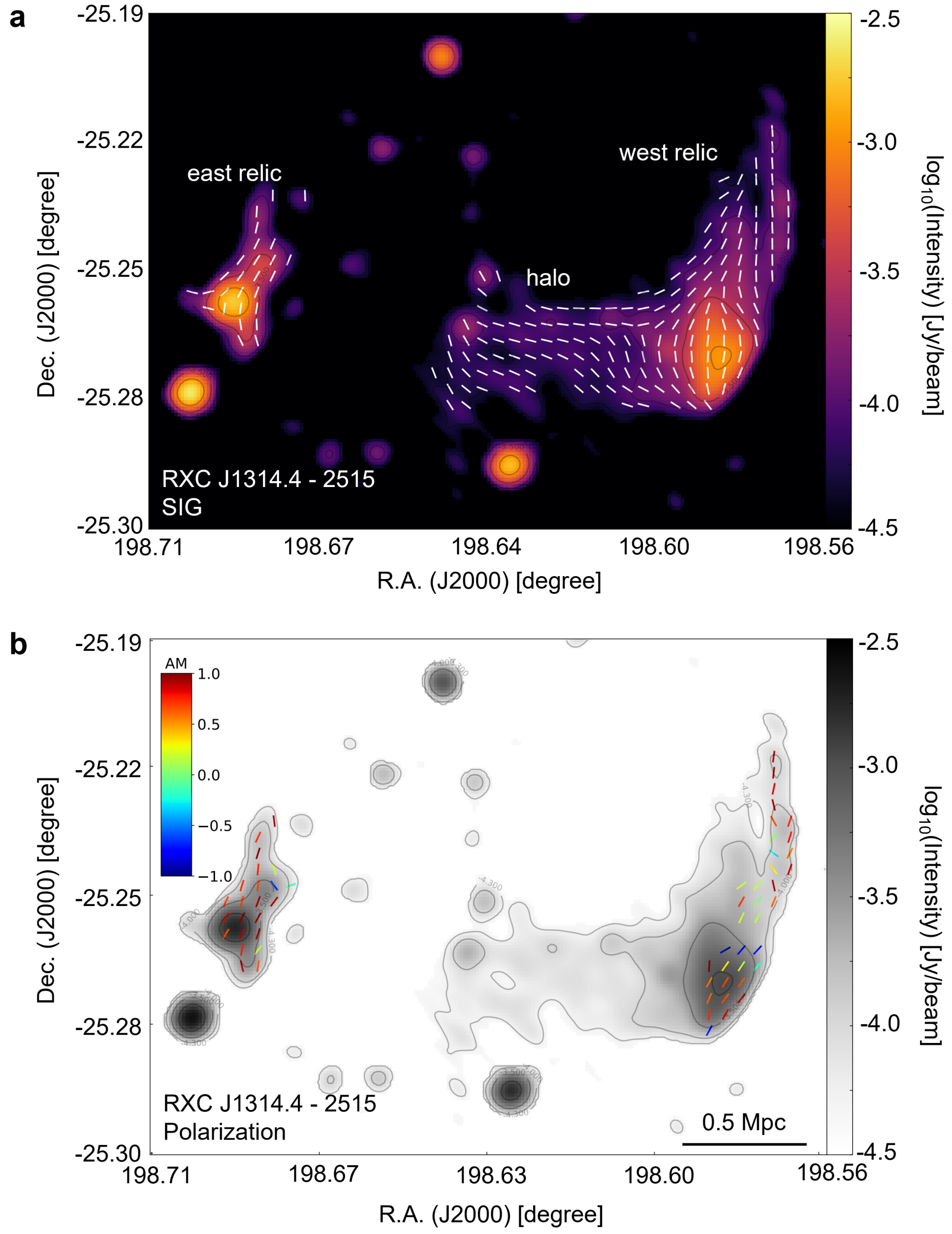

Supplementary Figure 1. The magnetic field orientation of the RXC J1314.4 - 2515 cluster. Panel a: the magnetic field mapped by the Synchrotron Intensity Gradient (SIG) technique with an FWHM of approximately 240 kpc. Panel b: the magnetic field determined through synchrotron polarization at 3 GHz using the JVLA radio observations, with an FWHM of approximately 120 kpc. The magnetic field is overlaid on the synchrotron emission intensity map, with colors indicating the AM between the SIG and polarization. Each (magnetic field) segment represents the SIG (or polarization) averaged for 6×6 pixels for visualization purposes. Source data are provided as a Source Data file.

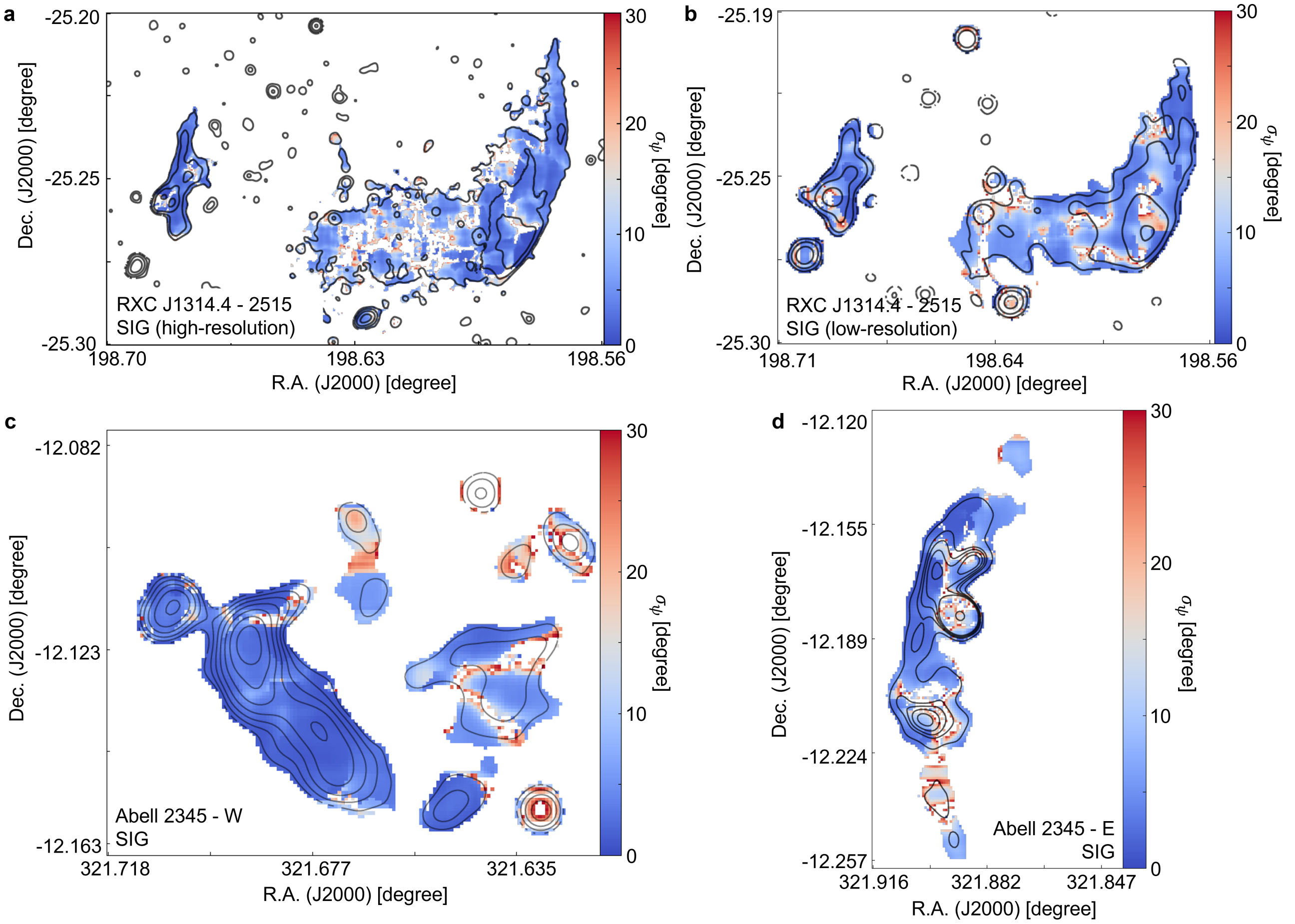

Supplementary Figure 2. Uncertainty maps for the magnetic field measured by the SIG for RXC J1314.4 - 2515 and Abell 2345 clusters. Panels a and b: RXC J1314.4 - 2515's uncertainty maps calculated from high-resolution (panel a) and low-resolution (panel b) observations. Panels c and d: Abell 2345's uncertainty maps calculated for Abell 2345 - W (panel c) and Abell 2345 - E (panel d) observations. Source data are provided as a Source Data file.

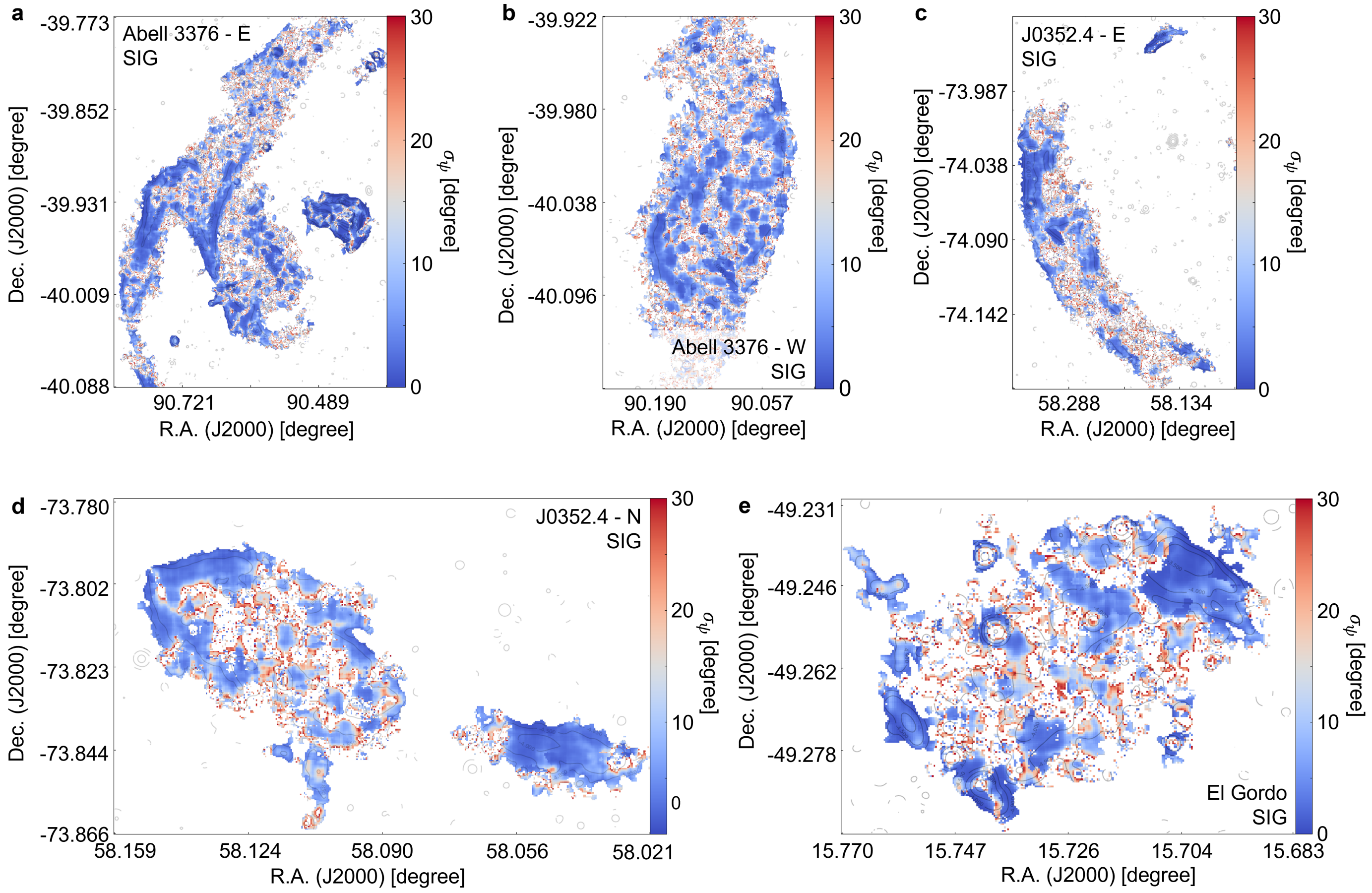

Supplementary Figure 3. Same as Supplementary Fig. 2, but for the cluster Abell 3376 (panels a and b), MCXC J0352.4 - 7401 (panels c and d), and El Gordo (panel e). Source data are provided as a Source Data file.

the contribution from only one beam. Therefore, we expect that the contribution from turbulence or large-scale flows should dominate the gradient signal.

The relative angle between the POS magnetic field and merger-axis

Supplementary Figs. 4 and 5 present the relative angle between the POS magnetic field and the merger axis as a function of distance from the center of the cluster. The relative angle is determined by averaging over linearly spaced radial bins in annuli from the cluster center. A relative angle value of $> 45^\circ$ signifies that the magnetic field is primarily perpendicular to the merger axis, while a value of $< 45^\circ$ indicates that the magnetic field is predominantly parallel to the axis. The central coordinates for the five studied clusters are: [R.A.: 198.617°, Dec.: −25.261°] for RXC J1314.4 - 2515, [R.A.: 321.796°, Dec.: −12.159°] for Abell 2345, [R.A.: 90.354°, Dec.: −39.998°] for Abell 3376, [R.A.: 58.086°, Dec.: −74.001°] for MCXC J0352.4 - 7401, and [R.A.: 15.718°, Dec.: −49.249°] for El Gordo. The merger axis, determined by the cluster center and the midpoint of the most prominent relic's longer axis, has orientations (north through east) of approximately 80°, −70°, 78°, −30°, −50° for RXC J1314.4 - 2515, Abell 2345, Abell 3376, MCXC J0352.4 - 7401, and El Gordo, respectively. To account for potential uncertainties, we repeat the analysis by rotating the merge axis by ±45°. However, the merger axis can also be different when derived from the X-ray map elongation or the optical analysis of the merging sub-clusters. Here we further analyze the merger axis determined by the X-ray coutour's elongation. The (X-ray) merger axis orients (north through east) approximately 90°, −15°, 78°, −30°, −30° for RXC J1314.4 - 2515, Abell 2345, Abell 3376, MCXC J0352.4 - 7401, and El Gordo, respectively.

The results suggest that magnetic fields in the relics are primarily perpendicular to the merger axis. However, in RXC J1314.4 - 2515 and EL Gordo, the magnetic field in the radio halos is preferentially parallel to the axis within a distance of approximately 0.5 Mpc. As a final note, these correlations are obtained for the POS magnetic field and projected merger axis.

The structure function of POS magnetic field orientation

Supplementary Fig. 6 presents the structure-function ($\mathrm{SF}_{\theta_\mathrm{r}}$) of the POS magnetic field orientation in the five clusters RXCJ1314.4 - 2515, Abell 2345, MCXC J0352.4 - 7401, Abell 3376, and El Gordo. The structure-function is defined as:

$$\mathrm{SF}_{\theta_\mathrm{r}}(l) = \langle(\psi(\boldsymbol{r}) - \psi(\boldsymbol{r}+\boldsymbol{l}))^2\rangle, \quad (6)$$

where $\psi(\boldsymbol{r})$ is the magnetic field orientation and $\boldsymbol{r} = (x, y)$ is the spatial position on the POS. We can see that, for Abell 2345, the structure functions of SIG and polarization are flat at large scales and the statistics of SIG are similar to those of polarization. For RXCJ1314.4 - 2515, polarization exhibits more significant angle fluctuations than SIG in the relics (scales larger than > 0.3 Mpc). The difference primarily comes from the less ordered magnetic fields (inferred from polarization) in the west-relic's north tail. The fluctuation of the magnetic field in the radio halo increases further. Generally, for the five clusters, the structure functions are flat on scales larger than > 0.3 Mpc.

References

1. Banfi, S., Vazza, F. & Wittor, D. Shock waves in the magnetized cosmic web: the role of obliquity and cosmic ray acceleration. *MNRAS* **496**, 3648–3667 (2020).

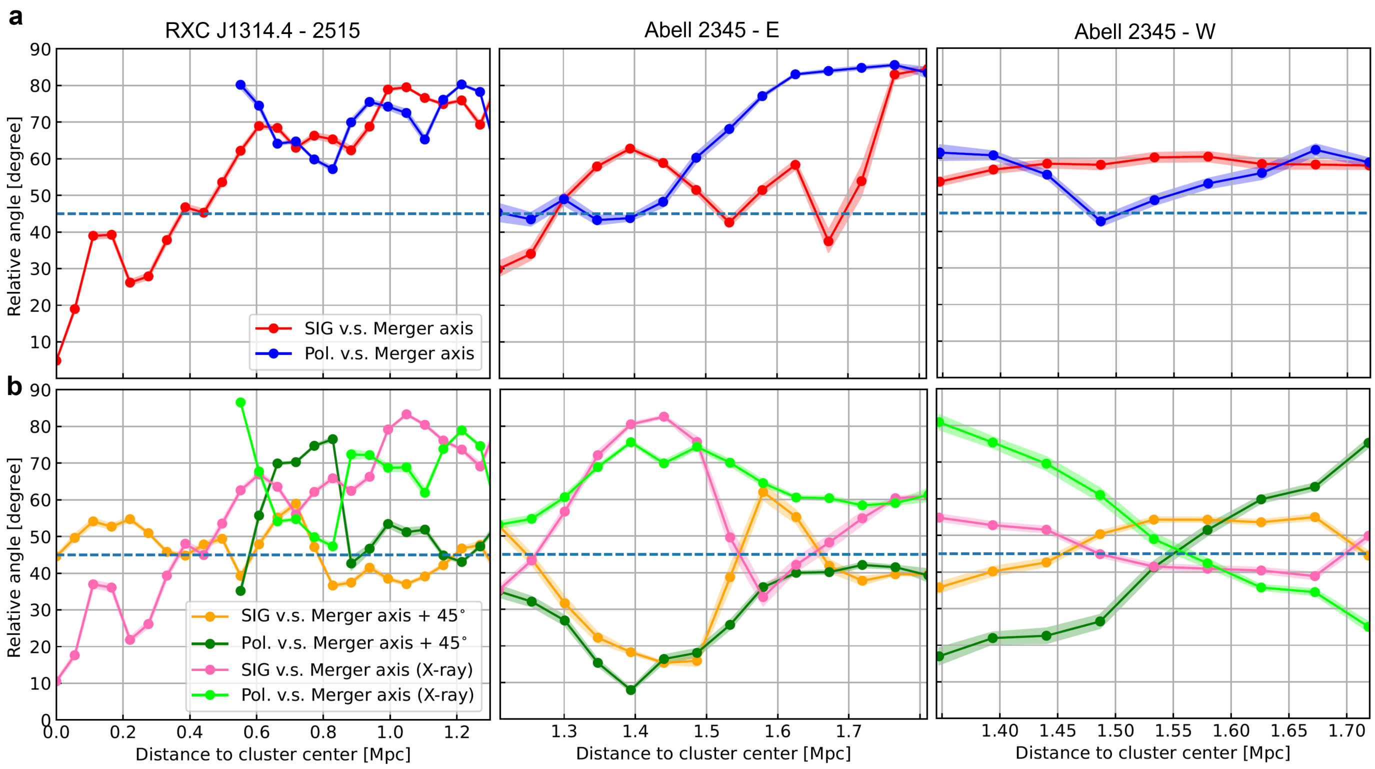

Supplementary Figure 4. Panel a: the relative angle between the POS magnetic field and merger-axis (determined by radio observation) as a function of the distance (x-axis) to the cluster center towards the RXCJ1314.4 - 2515 (FWHM approximately 120 kpc) and Abell 2345 (FWHM approximately 180 kpc) galaxy clusters. The relative angle $> 45^\circ$ indicates a preferentially perpendicular configuration, while $< 45^\circ$ suggests a parallel one. The dashed line presents that the relative angle is 45°. The shallower area represents the uncertainty calculated from the standard deviation. Panel b: same as panel a, but the merger axis (derived from radio data) is rotated by $\pm 45^\circ$ or is determined by the X-ray map's elongation. Source data are provided as a Source Data file.

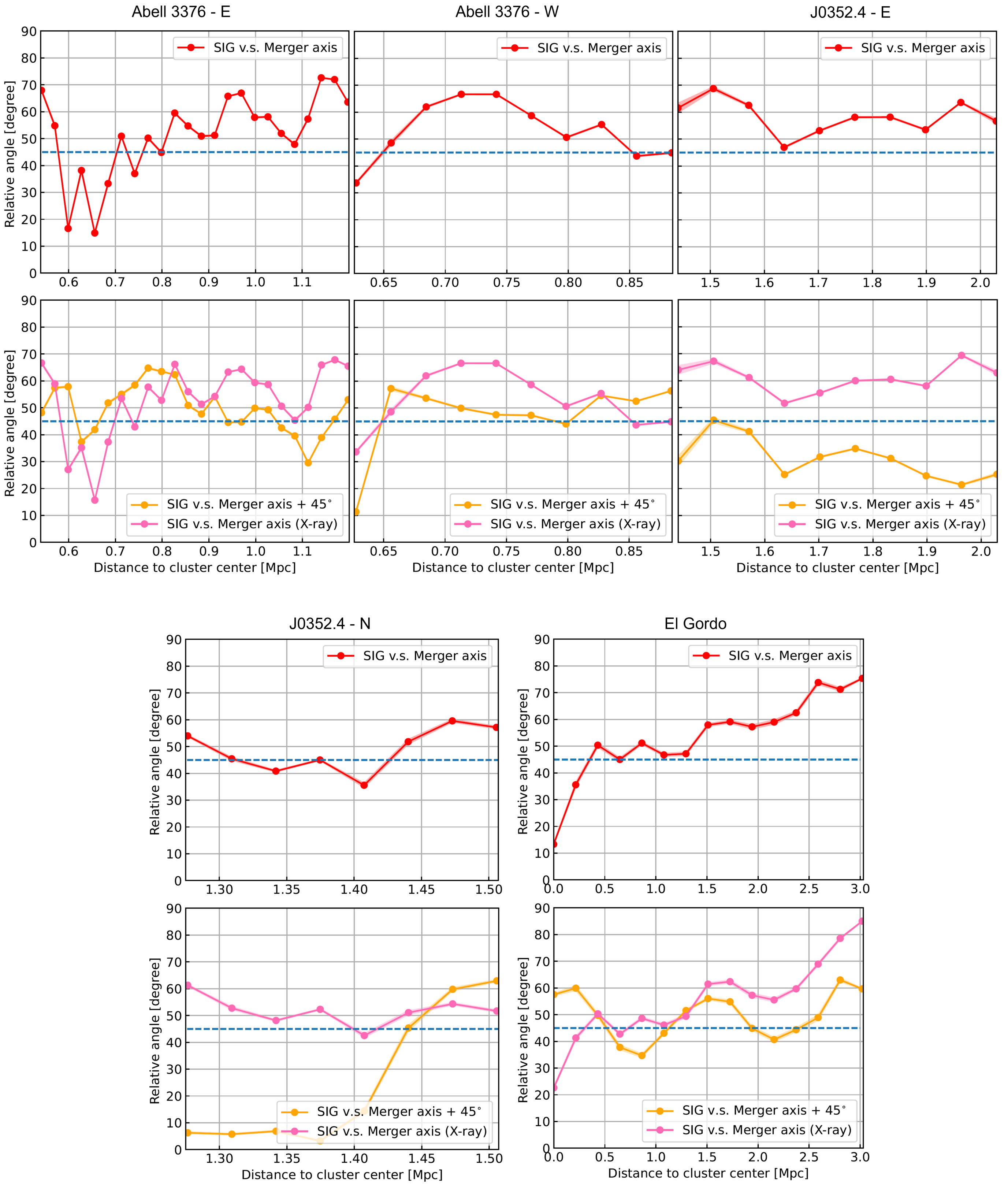

Supplementary Figure 5. Same as Supplementary Fig. 4, but for the clusters MCXC J0352.4 - 7401, Abell 3376, and El Gordo. The dashed line presents that the relative angle is 45°. Source data are provided as a Source Data file.

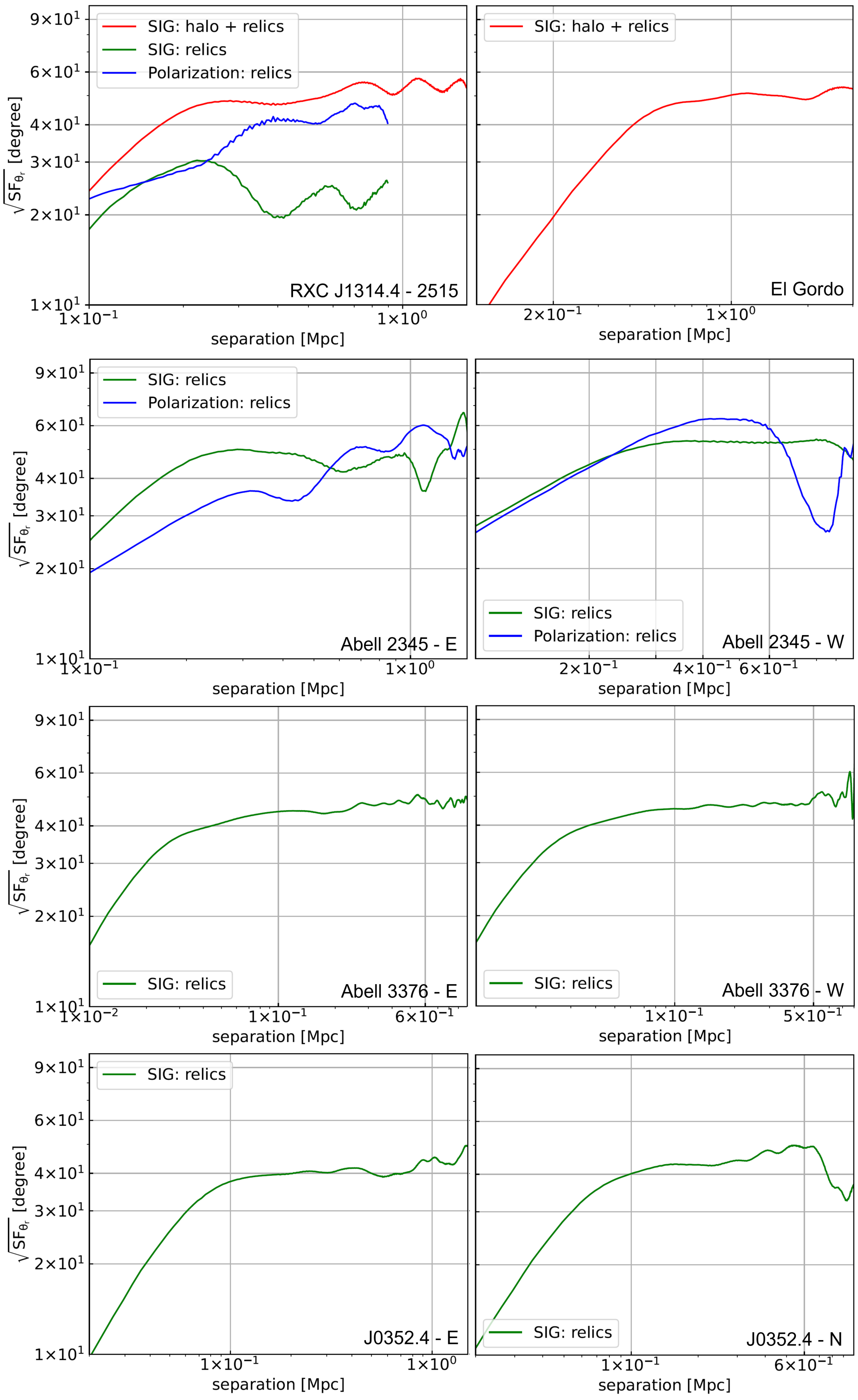

Supplementary Figure 6. The structure function of the POS magnetic field orientation in five clusters: RXCJ1314.4 - 2515, Abell 2345, MCXC J0352.4 - 7401, Abell 3376, and El Gordo. Source data are provided as a Source Data file.
